# Study on the Physico-Chemical Properties of the Si Nanowires Surface

**DOI:** 10.3390/nano9060818

**Published:** 2019-05-30

**Authors:** Rosaria A. Puglisi, Corrado Bongiorno, Giovanni Borgh, Enza Fazio, Cristina Garozzo, Giovanni Mannino, Fortunato Neri, Giovanna Pellegrino, Silvia Scalese, Antonino La Magna

**Affiliations:** 1 Istituto per la Microelettronica e Microsistemi, Consiglio Nazionale delle Ricerche, Strada VIII n.5, Zona Industriale, 95121 Catania, Italy; corrado.bongiorno@imm.cnr.it (C.B.); cristina.garozzo@imm.cnr.it (C.G.); giovanni.mannino@imm.cnr.it (G.M.); giovanna.pellegrino@imm.cnr.it (G.P.); silvia.scalese@imm.cnr.it (S.S.) antonino.lamagna@imm.cnr.it (A.L.M.); 2 Dipartimento Scienze Matematiche ed Informatiche, Scienze Fisiche e Scienze della Terra, Università degli Studi di Messina, Viale F. Stagno d’Alcontres, 31, 98166 Messina, Italy; giovanni_borgh@hotmail.it (G.B.); enfazio@unime.it (E.F.); fneri@unime.it (F.N.)

**Keywords:** nanowires, catalytic growth, etching, gold, VLS, CVD

## Abstract

Silicon nanowires (Si-NWs) have been extensively studied for their numerous applications in nano-electronics. The most common method for their synthesis is the vapor–liquid–solid growth, using gold as catalyst. After the growth, the metal remains on the Si-NW tip, representing an important issue, because Au creates deep traps in the Si band gap that deteriorate the device performance. The methods proposed so far to remove Au offer low efficiency, strongly oxidize the Si-NW sidewalls, or produce structural damage. A physical and chemical characterization of the as-grown Si-NWs is presented. A thin shell covering the Au tip and acting as a barrier is found. The chemical composition of this layer is investigated through high resolution transmission electron microscopy (TEM) coupled with chemical analysis; its formation mechanism is discussed in terms of atomic interdiffusion phenomena, driven by the heating/cooling processes taking place inside the eutectic-Si-NW system. Based on the knowledge acquired, a new efficient etching procedure is developed. The characterization after the chemical etching is also performed to monitor the removal process and the Si-NWs morphological characteristics, demonstrating the efficiency of the proposed method and the absence of modifications in the nanostructure.

## 1. Introduction

Silicon nanowires (Si-NWs) have drawn increasing attention for decades because of their notable electrical and optical properties [1,2]. An important advantage of their geometry relies on the fact that Si-NWs are suitable for making electronic devices based on the 3-dimensional architectures, nowadays considered promising systems to reduce the costs and increase the performance. In the photovoltaic sector, for example, they have been indicated as a potential solution to improve the efficiency/cost ratio of the solar cells for a number of reasons: They allow decoupling of the photon absorption from the carrier collection paths through the radial p–n junction [3]; increase the generation rate of excitons through light trapping mechanisms, making the coupling with radiation more efficient [4,5,6]; decrease the thermalization losses, owing to the band gap modulation for the quantum size effect, thus converting more efficiently the blue photons into electrical energy [7].

One of the most widespread methods used to synthesize Si-NWs is the chemical vapor deposition (CVD), which uses a metal cluster catalyst to induce the growth [2,8,9,10,11,12,13,14,15,16,17]. In particular, among the several types of CVD systems, the plasma-based ones are more promising because they spend lower thermal budgets compared to the others, as well as allow accurate control of process parameters, a fundamental aspect in industrial production [4,18,19,20]. For this reason, they are already present in the semiconductor fabs. Other CVD advantages are the atomically flat surface, the very high aspect ratios, and few crystallographic defects. 

The process based on the catalyzed growth is known as vapor–liquid–solid (VLS) [21], and it is one of the most known metal-induced processes for Si-NWs growth. A liquid eutectic alloy droplet composed of a blend of metal and Si is first formed on the silicon wafer surface that acts as a preferential site for the adsorption of Si precursor, such as SiH_4_. The Au–Si drops indeed significantly lower the activation energy as they adsorb the precursor directly from the gaseous phase, accelerating the growth compared to the classic vapor–solid process (VS). Then the metal–Si alloy acts as a true catalyst, it selectively continues to adsorb Si vapor above supersaturation limit, triggering its precipitation and launching the nucleation of Si seeds at the solid–liquid interface promoting the Si-NWs growth. The growth proceeds in definite crystallographic directions dependent on that of the substrate surface orientation or on their diameter [22]. By stopping the steam flow of the precursor, the VLS growth ends; however, metallic seeds remain linked to the top of the NW tip. The most commonly used catalyst for Si-NWs growth is gold for the many advantages that it presents such as, a low eutectic temperature with silicon (363 °C), fast interdiffusion of the two atoms Au and Si, high solubility allowing the formation of Au_82_Si_18_ through a deep reaction [23], and the opportunity to easily form liquid alloys with the growth precursor.

Despite of its advantages, there are some relevant issues related to the use of Au as a catalyst. In fact, it is well-known that gold is highly detrimental to the performance of minority carriers, because it easily introduces alterations in the system electronic structure. In particular, it generates a deep acceptor state at Ec–0.54 eV in n-type Si and a donor level at Ev–0.35 eV in p-type Si that act as effective recombination centers [24], reducing the minority carrier lifetime, consequently causing the electronics devices electrical and optical performance degradation [25,26,27,28,29]. Indeed, Perraud et al. [30] found that the value of the reverse current in the p–n junction of their Si nanowire array solar cell, fabricated by Au-induced growth and without Au removal process, was on the order of 1 μA/cm^2^, which is extremely high compared to that of a typical silicon wafer solar cell (i.e., 1 pA/cm^2^) and they attributed this effect to the presence of the Au. Therefore, it is unavoidable to make a further removal step of the Au at the tip of the Si-NWs.

In addition to the wire tip, gold can also penetrate inside the nanostructure. The inclusion of metal catalyst atoms into the growing NWs has often been observed during the VLS growth mechanism; with the use of advanced analytical methods [9,28,31] it was demonstrated that the maximum Au concentration can span between 1.7 × 10^16^ cm^−3^ [9] and 5 × 10^17^cm^−3^ [28]. 

The harmful properties of gold have motivated several works on the optimization of the gold removal after the nanowires growth by VLS [9,32,33]. These methods, however, create structural damages on the Si-NWs structure, strongly oxidize the Si-NWs surface, or require long processing time (in some cases up to 24 h). Strong selectivity for Au, low damage on the Si-NW walls, short processing times, high efficiency, inexpensiveness, and simplicity are the features that should be guaranteed by an ideal removal process. To achieve them, a deep understanding of the physical and chemical characteristics of the Si-NWs surfaces and reactivity is necessary and needs to be first pursued. The reason for the poor removal efficiency of the methods proposed is suggestive of the existence of a shell that surrounds the Au seeds [34,35,36], creating a barrier for the chemical etchant [32,37,38]. Hessel and coworkers proposed a rapid thermal quenching of the nanorods that creates cracks or defects in the shell and allows the etchant to reach the inner Au dot [32].

The presence of the shell surrounding the Si-NWs was observed by several other research groups [39,40,41,42], but the reason behind its formation is not yet completely clear. A possible cause has been indicated in the weakness of the covalent bond of the silicon atoms at the interface with the gold that would remove them from the crystal lattice and let them spread through the gold dot toward its external surface, thus continuing to accumulate at the Au/air interface. This thin silicon layer then oxidizes after its exposure to air [34,40]. It is known than the gold diffusion in Si, after the kick-out mechanism in interstitial sites, is influenced by the supersaturation of Si point defects (vacancies and self-interstitials) [43]. The fast diffusion could be related to the electron configuration of the Au atoms, because they have only 1s valence electron with fully complete other shells. Another work in literature specifically focuses on the phase separation step of Au–Si eutectic after the growth, discussing the system cooling process during which Si atoms segregate forming a thin Si film on the surface of the catalyst and the successive air exposure results in its oxidation [34]. This SiO_2_ coat was also found at temperatures well below the Si–Au eutectic point (T = 150–200 °C) [44], thus indicating a low temperature threshold for its formation.

Sivakov and coworkers found that Au atoms agglomerate on Si-NWs sidewall after an annealing of about 800 °C for 0.5 h without any previous etch process [45]. This suggests that if some residual gold is still present in the material even after the removal process, the annealing could allow Au atoms to undergo ripening and agglomerate on Si-NW sidewalls, allowing an easier revelation.

In this work, deep study on the physico-chemical properties of the surface of the Si-NWs grown by Au-mediated VLS is presented. The characterization was focused on the understanding of the chemical composition of the Au tip shell and on its formation mechanisms. Based on these studies, an optimized process for the gold removal from Si-NWs is developed. It consists of the combination of two separate wet etching steps: selective removal of the Au shell and subsequent gold chemical etching. The Si-NWs characterization, in terms of their morphology, density, surface chemical composition, is presented.

The Si-NWs structural and morphological characteristics are investigated through scanning electron microscopy (SEM) and transmission electron microscopy (TEM), before and after the chemical etching. The composition of nanowires surface has been deeply characterized by energy filtered TEM (EFTEM) and XPS measurements.

To evaluate the effectiveness of the two-step procedure on the Si-NW sidewalls, we have performed an annealing at 800 °C in order to allow the agglomeration of the possible residual Au atoms [20]. The tested method is fully adaptable with integrated circuit technology and is compatible with all the Si-NWs synthesis techniques where Si-NWs are grown using Au as catalyst.

## 2. Materials and Methods

The substrate used for the Si-NWs growth was <100> p type 6" Si wafer. After a brief etch in hydrofluoric (HF) acid, the Au dots with density of 2 × 10^12^ cm^−2^ and an average radius of 1.6 nm were deposited by sputtering, and after a second HF dip they were loaded into the CVD chamber. The second HF dip was performed to remove the thin silicon oxide shell present on the surface of the gold nanodots, and to promote catalysis [40]. The whole substrate preparation was performed in sequence. The substrates were loaded into the plasma-assisted CVD chamber and heated at 380 °C for 1 h before the growth. Si-NWs were grown at 380 °C with a deposition time of 15 min using a gas ratio of SiH_4_/Ar = 30, and plasma power of 20 W. After VLS growth, Si-NWs underwent a two-step procedure allowing the Au removal: First the sample was immersed for 5 min in a HF buffered solution diluted in H_2_O (1:10) in order to allow the SiO_2_ shell removal; and subsequently in a gold etch solution (Fujifilm gold etch II w/OHS), containing sodium iodide (NaI) and iodine (I_2_), diluted in H_2_O (1:10) for 4 min. The whole etching process lasts 9 min. Some samples, instead, underwent a single etching step with the gold etch solution without the HF pretreatment and served as a comparison. After the etching, some samples were annealed at 800 °C in N_2_ to allow agglomeration of the possible residual Au atoms. All the samples have been analyzed by using a Zeiss Supra35 scanning electron microscope (SEM) with a field emission electron gun, and by transmission electron microscopy (TEM) equipped with a system for energy filtering (EFTEM). The NWs density after every step of etching was evaluated by counting NWs directly from the SEM micrograph in planar view. After a proper calibration of the SEM micrographs, the diameter of the NWs was measured using a tool of Image Pro Plus.

Accurate analysis of the possible presence of gold after etching was performed by means of TEM in scanning mode (STEM) with a JEOL 2010F TEM/STEM system equipped with a 200 kV field-emission electron gun, and by X-ray photoelectron spectroscopy (XPS) analyses, which were recorded with a PHI ESCA/SAM 5600 Multy technique spectrometer equipped with monochromatized Al Ka X-ray source. The XPS analyses were performed at a take-off angle of 45°. The binding energy (BE) scale was calibrated by centering the C 1s signal of the adventitious carbon at 285.0 eV.

## 3. Results and Discussion

**Figure 1:**
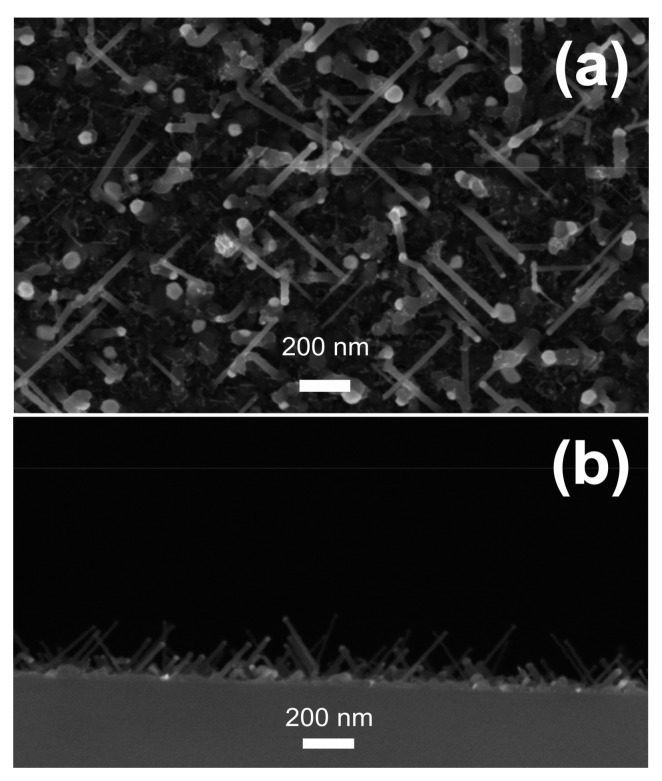
Scanning electron microscope (SEM) micrographs in planar (**a**) and cross-sectional view (**b**) of silicon nanowires (Si-NWs) array grown on Si (100) substrate, prepared by Au-catalyzed VLS growth in plasma ambient.

SEM micrograph in Figure 1a shows the typical top-view image of the sample after NWs growth. The light grey sphere on the top of the nanowires represents the gold catalyst residual. Figure 1b shows the cross section of the SEM image for the same sample as in Figure 1a. The diameter of the NWs is constant along the length. It is known that NWs grown by CVD using Au as catalyst tend to preferentially grow with three directions with respect to the (100) substrate, i.e., <100>, <112>, and <111> depending on the NWs diameter, that match to 54°, 45°, and 18.4°, respectively [46]. The slanting orientation of nanowires allows for the direct measurement, in planar view, of the key parameters such as diameter and density with a good statistics. Therefore, the morphological characteristics were monitored before and after Au removal from the substrates in order to evaluate the effect of chemical etching on the Si-NWs surface density.

**Figure 2:**
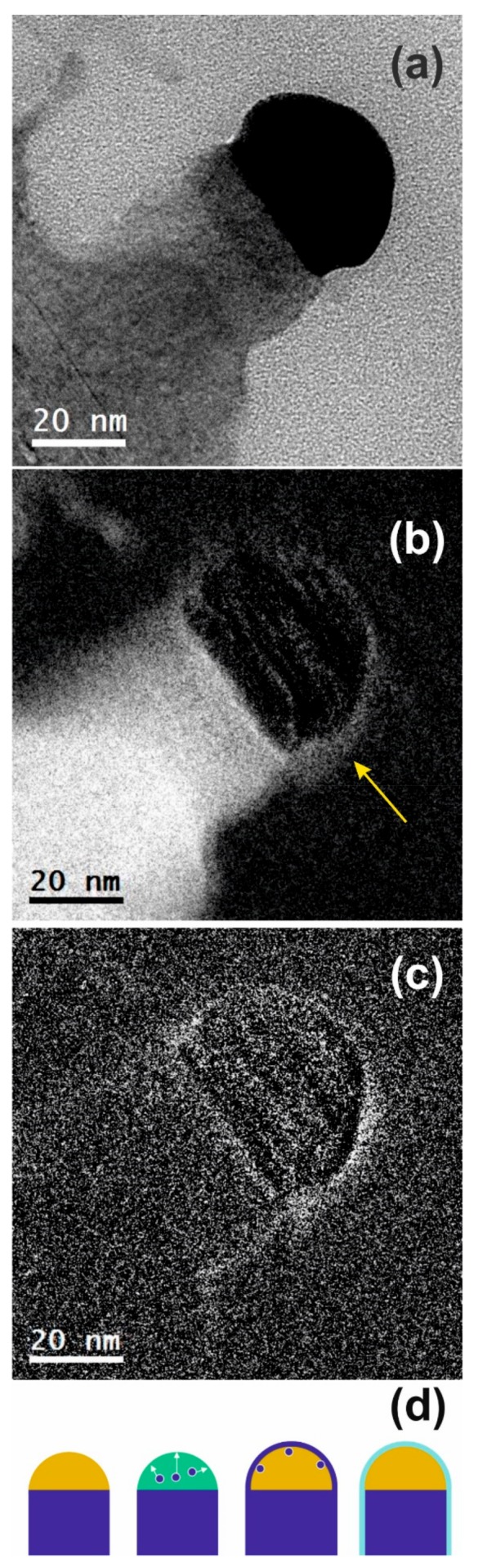
Transmission electron microscopy (TEM) micrograph acquired in bright field conditions of a Si-NW on <100> Si substrate (**a**). Silicon map (**b**) and oxygen map (**c**) acquired through energy filtered TEM (EFTEM) analysis on the same nanowire. A thin layer (indicated by the yellow arrow in **b**) is observed around the metal dot. Schematics illustrating the mechanisms of SiO_2_ layer formation around the Si-NWs tip (**d**).

Figure 2a shows a bright field TEM micrograph of an as-grown Si-NW, with a diameter of about 28 nm. The black hemisphere on the Si-NW tip represents the gold catalyst dot. It is evident that no Au dots are present on the Si-NW sidewall, contrary to what was reported in some other works [47]. In that case, Au agglomeration was attributed to the presence of a thin layer of gold covering the Si-NWs formed because of the high temperature of molecular beam epitaxy process (525 °C), which disintegrates in tiny gold clusters during final cooling. The low growth temperature (380 °C) and high growth rate obtained (about 17 nm/min) in our system could explain the absence of gold dots on the Si-NWs sidewalls. Moreover, the lower thermal budget reduces the gold diffusion into nanowires core and into the bulk. Literature data [48] indicate that if we consider Au diffusion in bulk silicon at 380 °C for the entire process time, pre-growth and post-growth (75 min), we expect the diffusion length in our sample to be about 25 nm; a short distance compared to the literature data of nanowires grown using 650 °C for 90 min, in which we expect a diffusion length greater than 2 μm [49]. This may suggest that if we do not detect the gold, the reason is that it is not present, not because it did not have enough thermal budget to diffuse. 

Ressel and coworkers studied the behavior of Au–Si liquid alloy on silicon substrate after an annealing process at temperatures higher than the eutectic one, and they found that Si is dissolved in the droplet during heating, precipitates out during the cooling process, and covers isotropically the catalyst dot. After gold etching they demonstrated the presence of an empty Si shell that encapsulates the solidified droplet [34].

The silicon map obtained by EFTEM analysis in Figure 2b highlights the presence of a thin layer (indicated by the yellow arrow) around the metal dot and on the Si-NWs sidewall. An oxygen map was also acquired in the same region to evaluate its chemical composition. The result shown in Figure 2c reveals that it is silicon dioxide. In Figure 2b,c, the integration effect during the TEM imaging on the lateral curvature of the investigated nanostructures due to their 3-dimensional nature, allows for the white signal identification on the sidewalls with a contrast higher than that of the center of the gold dot.

The mechanism of SiO_2_ layer formation around the Au tip after the NW growth is shown in the illustration (Figure 2d). Silicon atoms (blue dot) are present in the droplet (green hemisphere) at a temperature higher than the eutectic point. The phase separation that occurs in the eutectic during the cooling following the growth of the nanowires causes the isotropic precipitation of the silicon outside the droplet (see white arrows). Solidifying it generates a thin strongly reactive Si layer on the surface of the metal. The air exposure results in its oxidation, thus forming the SiO_2_ conformal coat surrounding the gold dot.

At this point, it is important to understand the reason why during the cooling the Si migrates toward the external surface of the droplet, instead of forming a buried core.

To explain this phenomenon we consider the kinetics of cooling before and after the growth. During the growth we have two heat fluxes: Q_in_, which is conduction heat flux coming from the Si substrate, because the heat source is in contact with the sample holder; and a dissipation heat flux Q_outA_, that is convection heat toward the outside of the nanowires. When the growth process is stopped, the cooling of the nanowires occurs by two processes, dissipation of Q_outA_ with the chamber through convective transfer, and dissipation with the substrate Q_outS_ ruled by a conductive transfer. Q_outS_ is more efficient than Q_outA_. As a consequence, the cooling and the subsequent solidification starts from the inner core of the droplet and moves toward the surface. For this reason, the silicon atoms tend to segregate toward the surface that is warmer than the inner core of the shell and finally precipitates out creating a silicon layer that surrounds the metal catalyst dot. After the growth, when the sample is exposed to the air, the Si atoms react with the oxygen producing a SiO_2_ layer. The thickness of the shell depends on the initial thickness of the precipitated Si layer, which in turn depends on the degree of supersaturation of Si in the initial Si/Au eutectic droplet. However, the thickness of SiO_2_ layer is not always constant, which could be due to local heat fluctuations.

The existence of this SiO_2_ shell is crucial for the chemical removal of metal seeds, because it acts as a passivating layer that impedes the physical access of chemical etchants. In this paper, we propose a new approach for the removal of the remaining gold catalyst from the Si-NWs using a two-step procedure. First, a pretreatment in a buffered solution of HF is done to remove the oxide shell, and second the gold is removed by using a gold etchant solution. 

**Figure 3:**
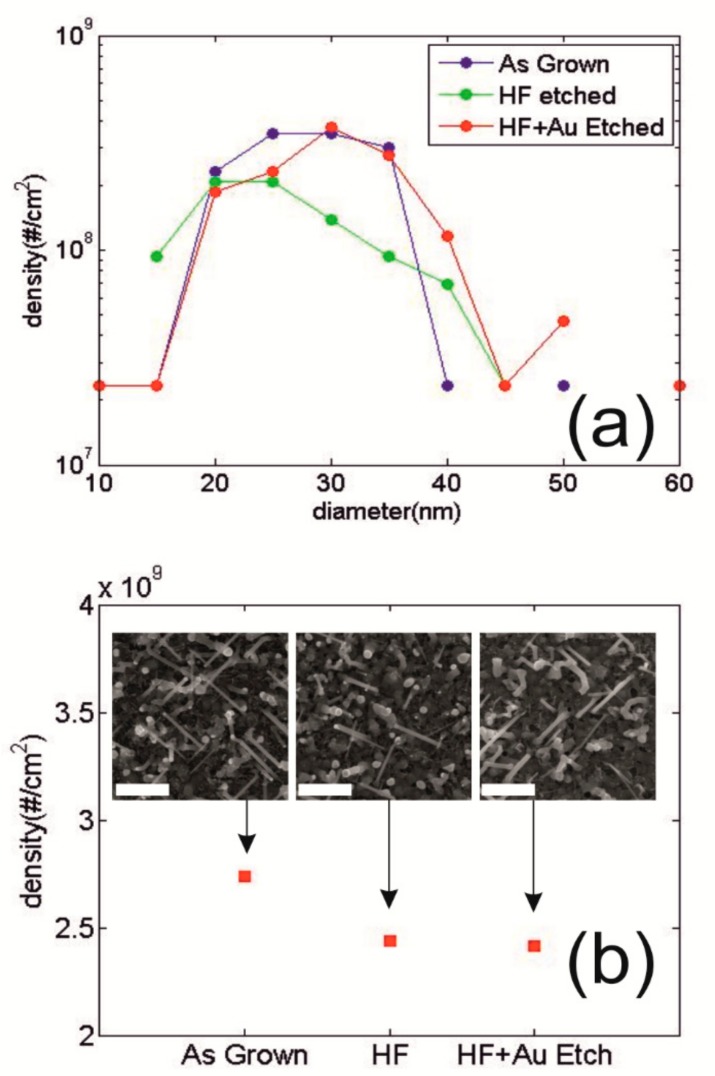
(**a**) Si-NWs density as a function of diameter is reported for as-grown sample (blue circles), after the HF etching (green circles) and after HF + Au etching (red circles). (**b**) Average Si-NWs surface density for the three cases under study. The inset shows the relative SEM micrograph in planar view scale markers corresponds to 400 nm.

The possible changes on the samples in terms of Si-NWs density and diameter distribution, have been evaluated after each step of etching using SEM analysis. The Si-NWs radius distribution has been measured by the digital analysis of the SEM images in planar view and on a large statistics. The results for the as-grown samples (blue circles), for the samples after the single etching step with HF (green circles), and after the double step with HF + Au etching (red cycles) are reported in Figure 3a. As it is evident from the curves, there is no significant difference between the distribution of the as-deposited case and after the etching steps, thus indicating that the chemical reaction does not produce damage in the samples.

It can be then stated that the morphology of the nanostructured surface is maintained unaltered after the process, fundamental aspect for the control of the final electrical features for efficient devices.

These results are supported by Figure 3b, which shows that the density slightly decreases after the HF etching, and remains almost constant after HF + Au etching. SEM images in planar view of the analyzed samples are also shown in the inset of Figure 3b.

The role of the HF pretreatment in the metal catalyst removal was assessed by comparing the structural characteristics of a sample that has undergone only the Au etching step and a sample subjected to the whole double-step procedure, i.e. the HF pretreatment and the Au etching step.

**Figure 4:**
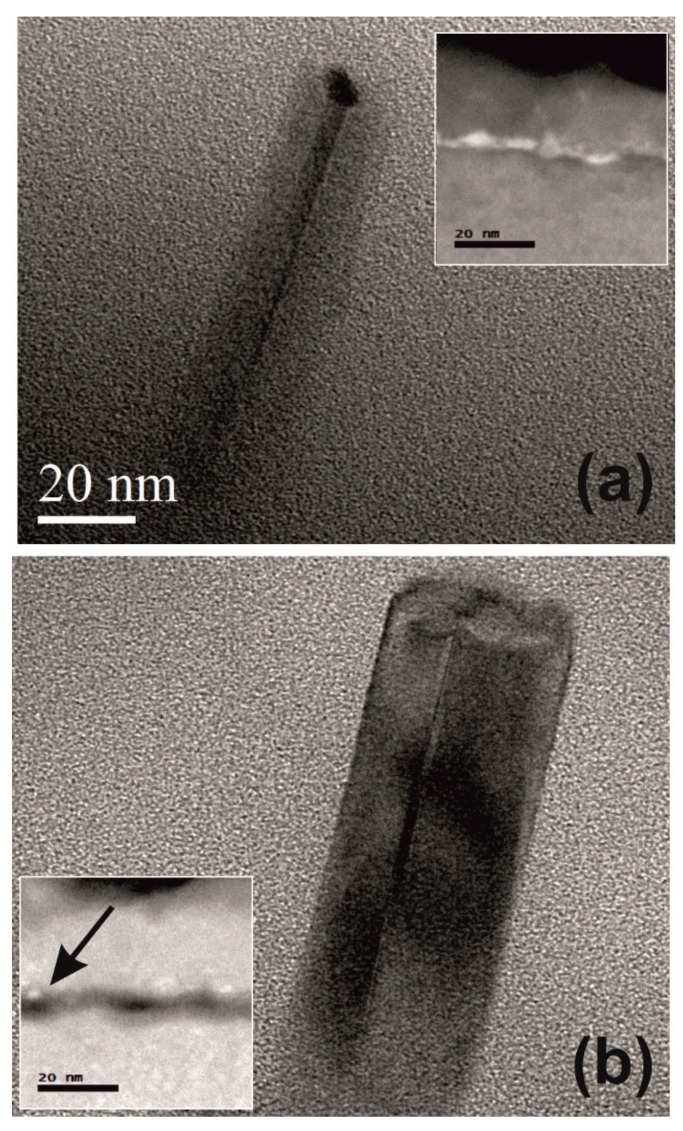
Cross-sectional view of TEM micrographs of Si-NWs after the gold removal single step (**a**), and after the two-step gold etching (**b**). Insets: STEM micrographs of the substrate/Si-NWs interface for the relative samples.

Figure 4a shows TEM micrograph of one nanowire after the gold removal step. The Au seed is just partially removed atop the Si-NW. The inset shows the STEM micrograph of the substrate/Si-NWs interface; it is evident that, an almost continuous white layer at the interface representing a thin layer of “buried gold nanodots” is present. They are not activated during the nanowires growth. The results of the samples that have undergone the complete two-step procedure are shown in Figure 4b. The TEM micrograph of the nanowire shows that no Au dot is detected after the two-step etching on the nanowires tip or on the nanowires sidewall, and no structural damage is observable on the nanostructure. The STEM studies of substrate/Si-NWs interface, shown in the figure inset, revealed that buried nanodots have been removed almost completely, and only in very rare occasions small Au nanodots were present in our sample as indicated by the black arrow. These nanodots are not easily removed because they are covered by a silicon layer that is not attacked by the combined HF and gold removing chemistry. This condition makes the gold removal less effective in the substrate/Si-NWs interface with respect to the NWs tip.

It is worth noting that no strong oxidation is visible on the Si-NW sidewall after the two-step Au etching process. An accurate TEM analysis has revealed the presence of a thin oxide shell on the wire sidewall with a size similar to the native oxide thickness. 

**Figure 5:**
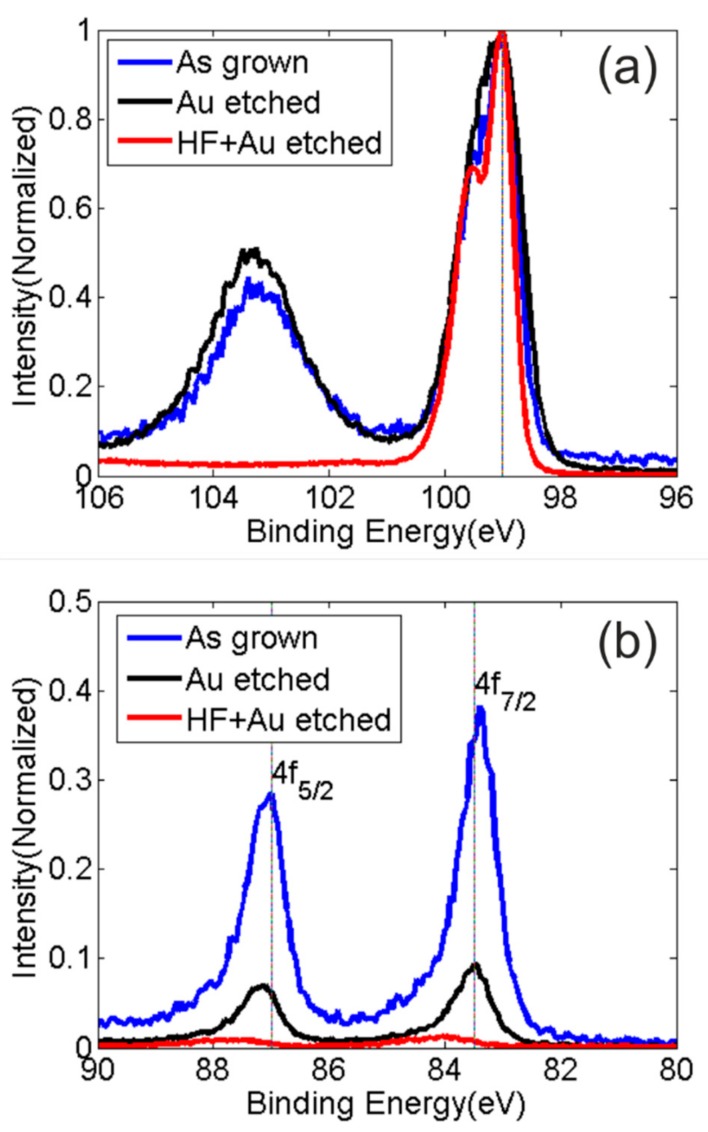
High resolution X-ray photoelectron spectra of the (**a**) Si 2p and (**b**) Au 4f of the as-grown nanowires (blue line), the sample obtained after the gold etching (black line) and the sample obtained after the two-step procedure (red line).

The modification of the NW surface composition as a consequence of the chemical processes has been followed by X-ray photoelectron spectroscopy (XPS). Owing to its quantitative accuracy, the technique is widely used in the field of solid state materials for the evaluation of the relative percentages of the elements at the surface. The high value of the atomic sensivity factor (A.S.F.) of the gold signal has allowed to monitor, with a good confidence, the presence of the metal after each procedure step. High resolution XPS spectra of the (a) Si 2p and (b) Au 4f of the as-grown nanowires (blue line), the sample obtained after the gold etching (black line), and the sample obtained after the two-step procedure (red line) are reported in Figure 5. The binding energy positions of the XPS peaks in the spectra are normalized by centering the C1s signal of the adventitious carbon at 285.0 eV [50]. The XPS Si 2p spectral region of all the samples exhibit a doublet originated by the spin-orbit splitting of the Si 2p3/2-1/2 (Si 2p3/2 = 99.0 eV) that is attributed to the elemental state of the silicon in the Si-NWs (Si0). A band centered at 103.5 eV is detected in the spectra of both the as-grown sample and in that obtained after the gold etching, revealing the presence of the oxidized silicon (SiO_2_). The SiO_2_/Si intensity ratio is quite comparable in the two spectra, thus indicating that the gold removal process does not affect the surface composition of the oxidized Si-NWs. The use of HF in the double step procedure (HF + Au-etching), allows to almost completely remove the SiO_2_ (as emerges from the Figure 5-red curve). Beside the changes in the spectral features of silicon, the effect of the etching procedures are noticeable in the amount of gold at the surface. The Au XPS region (shown in Figure 5) presents a well defined doublet due to the Au 4f 5/2 (83.4 eV) and 4f 7/2 (87.0 eV) features. In the figure, the comparison between the Au XPS signals of all the NW samples is shown. The gold intensities are normalized to the total Si 2p intensity accordingly, to the respective sensivity factors (with source Al ká, A.S.F. Au 4f = 5.24 and Si2p = 0.281). 

As it can be noted, the Au removal procedure performed on the as-grown (oxidized) Si-NWs leads to a significant decrease in the gold percentage (see Table 1). This is because the HF solution, removing the passivating shell of SiO_2_ around the nanowires tip, contributes to expose Au to the action of the gold-etching solution. It is deduced that the double-step process results to be more efficient than the single-step procedure: the XPS quantitative data confirm that the percentage of the gold at the surface after the (HF + Au etching) is below the 0.1% with respect to the Si 2p signal. 

Nevertheless, the presence of gold detected by X-ray photoelectron spectra (XPS) analyses refers to a few nanometers of the NW surface. Accordingly, as reported by Wagner et al., the sampling depth (~3λ, with λ as the attenuation length) estimated for the Au 4f contribution results to be ~5 / 6 nm [51]. 

To deeply analyze the effectiveness of the removal, special attention must be paid on the possible presence of gold buried inside the nanowire core. Sivakov demonstrated that an annealing at 800 °C in air is helpful to agglomerate gold atoms on the Si-NWs surface [45]. We then performed an annealing at 800 °C for 30 min in inert ambient (N_2_) to agglomerate the possible residual gold atoms in larger clusters and to make them visible to TEM analysis.

**Figure 6:**
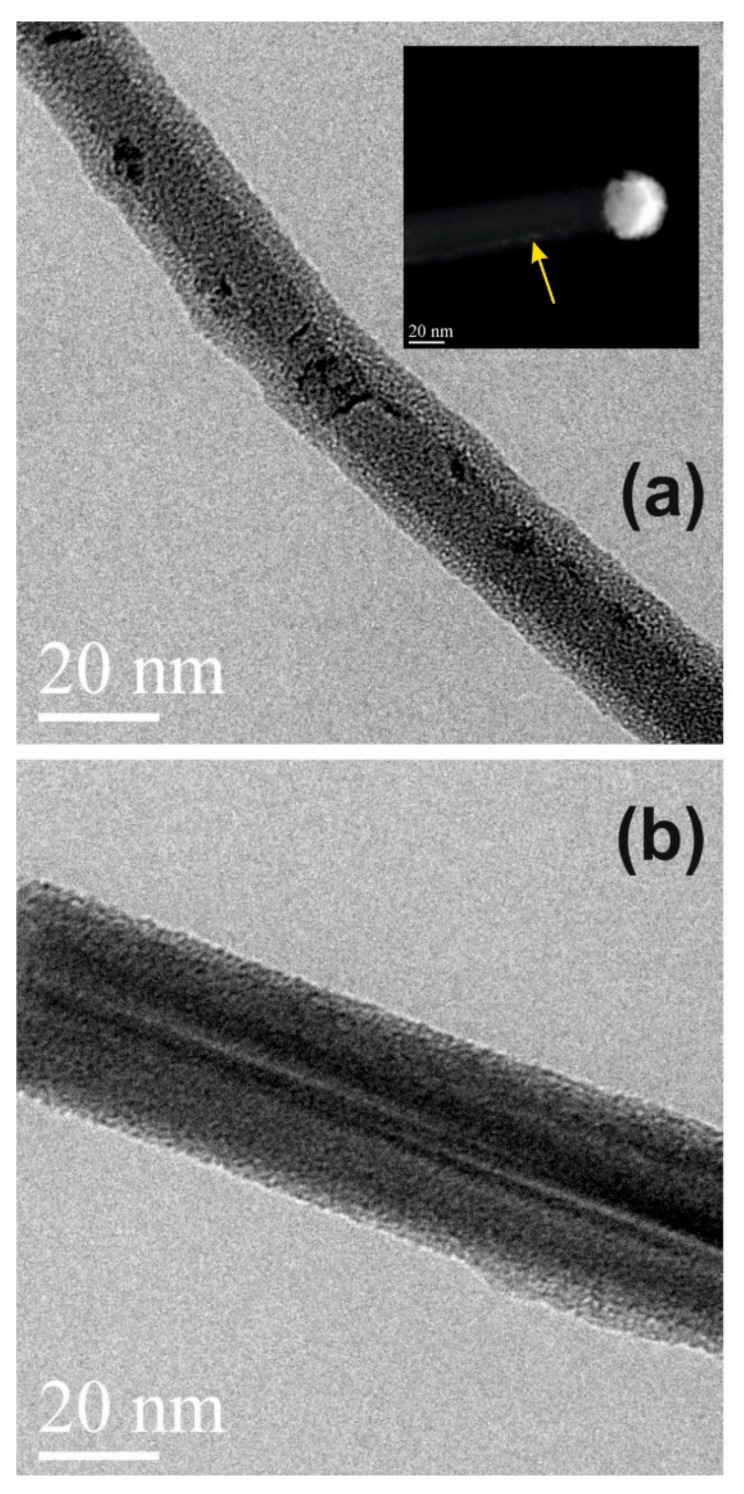
(**a**) TEM micrograph in cross-sectional view of nanowires without any previous gold etching process and annealed by Rapid Thermal Annealing (RTA) at 800 °C for 30 min. The inset shows a STEM image of the same sample. It is clearly visible that Au is present not only on the tip, but also on the NW sidewalls (as indicated by the yellow arrow). (**b**) TEM micrograph in cross-sectional view of Si-NWs with the double-step gold etching process and annealed by RTA at 800 °C for 30 min.

TEM micrograph in Figure 6a shows the sample annealed by RTA at 800 °C for 30 min without any previous gold etching process. Gold dots are present on the NW sidewalls presumably due to the agglomeration of gold atoms residual after the growth, thus confirming the effectiveness of the thermal annealing as a method for the detection of the Au residuals in the sample. A SiO_2_ shell over the NW surface is also present. Its formation is probably enhanced by the Au dot presence. The inset in Figure 6a shows a STEM image of the same sample in which it is clearly visible that Au is present not only on the tip, but also on the NW sidewalls (as indicated by the yellow arrow). Figure 6b shows the TEM micrograph of the sample after the complete gold removal procedure and subsequent RTA at 800 °C for 30 min in N_2_. It is possible to see that no Au nanodots are detected in the micrograph. However, the possible inclusion of Au catalyst atoms into the annealed nanowires cannot so far be excluded. Indeed, the solubility limit of Au in Si at 800 °C is about 10^15^ cm^−3^ [52]. Au atoms have not been detected after the process because the amount of inclusion is too low to be detected by the STEM, or because at 800 °C the thermal budget is not enough, in fact gold is a fast diffuser (10^−5^ to 10^−7^ cm^−1^·s^−1^) starting from 1100 °C [24].

## 4. Conclusions

In this work, the presence of the residuals of gold catalysts after the growth of Si-NWs by VLS is focused. The effectiveness of the most common chemical etching processes used so far are assessed, and their poor efficiency is attributed to the presence of an unwanted and conformal layer observed over Au dots, passivating the catalyst metal dots with respect to the action of the etchant solution.

A deep physical–chemical characterization has been performed on the as-grown Si-NWs, before any etching process, and on the conformal shell covering the Si-NWs tip and the shell composition is found to be oxidized silicon. The formation mechanisms of this shell are studied and its presence is attributed to the anisotropic kinetics of cooling in the Si–Au eutectic alloy after the Si-NWs synthesis; a positive thermal gradient is established during the cooling step from the bottom toward the top of catalyst dots. Consequently, silicon atoms segregate onto their surface forming an external silicon shell covering gold. This layer oxidizes after exposure to air generating an insulating silica thin film. A HF pretreatment is then proposed, before the Au etch, and the results of this double-step approach demonstrated an efficient and fast Au seed removal. XPS data confirm that the percentage of the gold at the surface is below 0.1% with respect to the Si signal. Moreover, the treatment does not produce damage on the Si-NWs in terms of density and walls corrosion, fundamental aspect to achieve efficient devices.

The possible presence of gold buried inside the nanowire core, even after the double-step procedure, has also been focused. Annealing at 800 °C has been performed to agglomerate possible residual gold atoms in Si-NWs. After the thermal process, no gold dots were found on the Si-NWs. 

## Figures and Tables

**Table 1:** X-ray photoelectron spectroscopy (XPS) atomic percentage of gold as revealed in the NW samples.

Relative Intensity	As Grown	Au Etching	HF + Au Etching
Au%	2.4	0.4	<0.1
